# Supplementary material for: Lignin-Loaded Carbon Nanoparticles as a Promising Control Agent against Fusarium verticillioides in Maize: Physiological and Biochemical Analyses
Source: Polymers (Basel). 2023 Feb 27;15(5):1193. doi: 10.3390/polym15051193 (PMC10007435; doi:10.3390/polym15051193)
Supplement: Supplementary file 1 [file polymers-15-01193-s001.zip › polymers-2199864-supplementary.pdf]

Supplementary Materials

Lignin- Loaded Carbon Nanoparticles: A Promising Control Agent Against *Fusarium verticillioides* in Maize: Physiological and Biochemical analyses

Sherif Mohamed El-Ganainy [1,2,3,*], Mohamed A. Mosa [4,*]; Ahmed Mahmoud Ismail [1,2,3] and Ashraf. E. Khalil [5]

[1] Department of Arid Land Agriculture, College of Agricultural and Food Sciences, King Faisal University, P.O. Box 420, Al-Ahsa 31982, Saudi Arabia; salganainy@kfu.edu.sa (S.M.E.-G.)
[2] Pests and Plant Diseases Unit, College of Agricultural and Food Sciences, King Faisal University, P.O. Box 420, Al-Ahsa 31982, Saudi Arabia; amismail@kfu.edu.sa (A.M.I.)
[3] Vegetable Diseases Research Department, Plant Pathology Research Institute, Agricultural Research Center (ARC), Giza 12619
[4] Nanotechnology & Advanced Nano-Materials Laboratory (NANML), Plant Pathology Research Institute, Agricultural Research Center, Giza 12619, Egypt; Email mohammed_sharouny@yahoo.com; (M. A. M.)
[5] Nematology Research Department, Plant Pathology Research Institute, Agricultural Research Center, Giza 12619, Egypt. Email: aashraf_373@yahoo.com ; (A.E.K)

Supplementary material S1: Schematic representation for the synthesis of lignin loaded carbon nanoparticles and their functional use in controlling *Fusarium verticillioides* in Maize

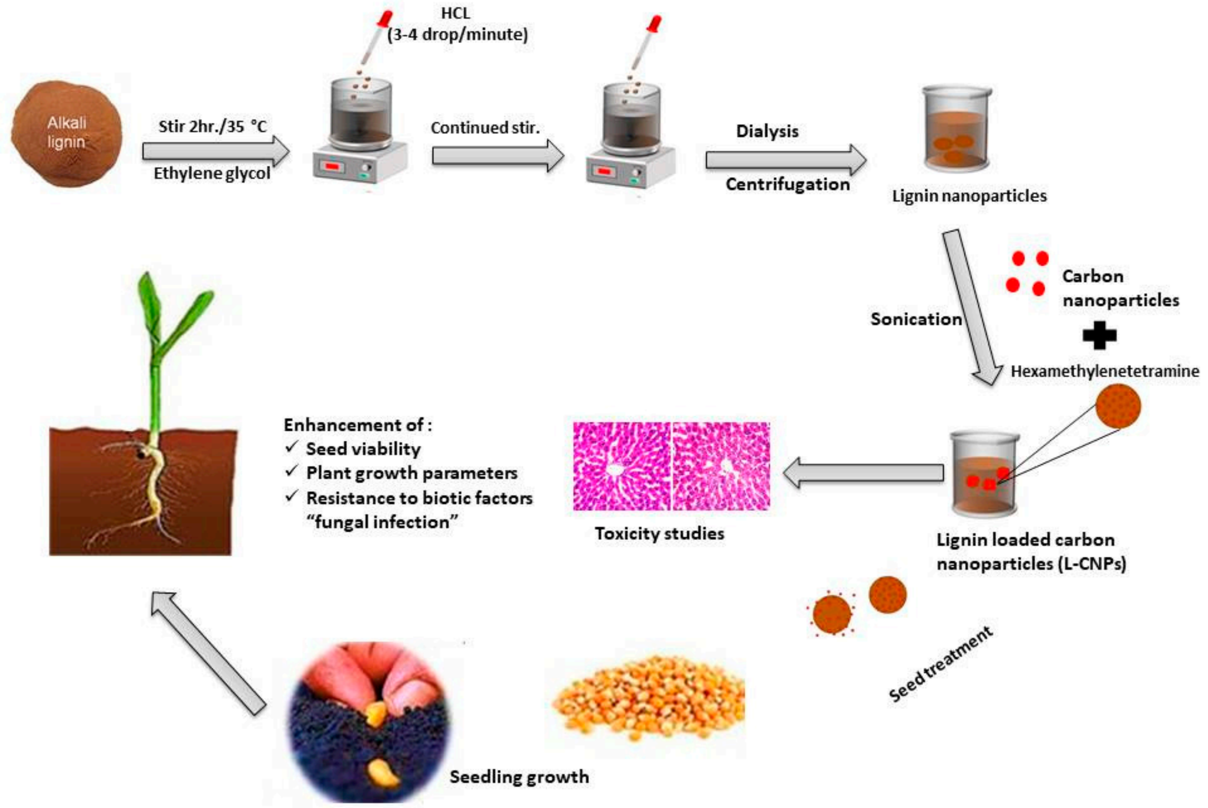

Figure S2. Dynmic light scattering (DLS) analysis of : (A) Lignin nanoparticles (L-NPs) at particle size (39.4 ± 2.1 nm) , and (B) Carbon nanoparticles (C-NPs) at particle size (15.3 ± 4.5 nm).

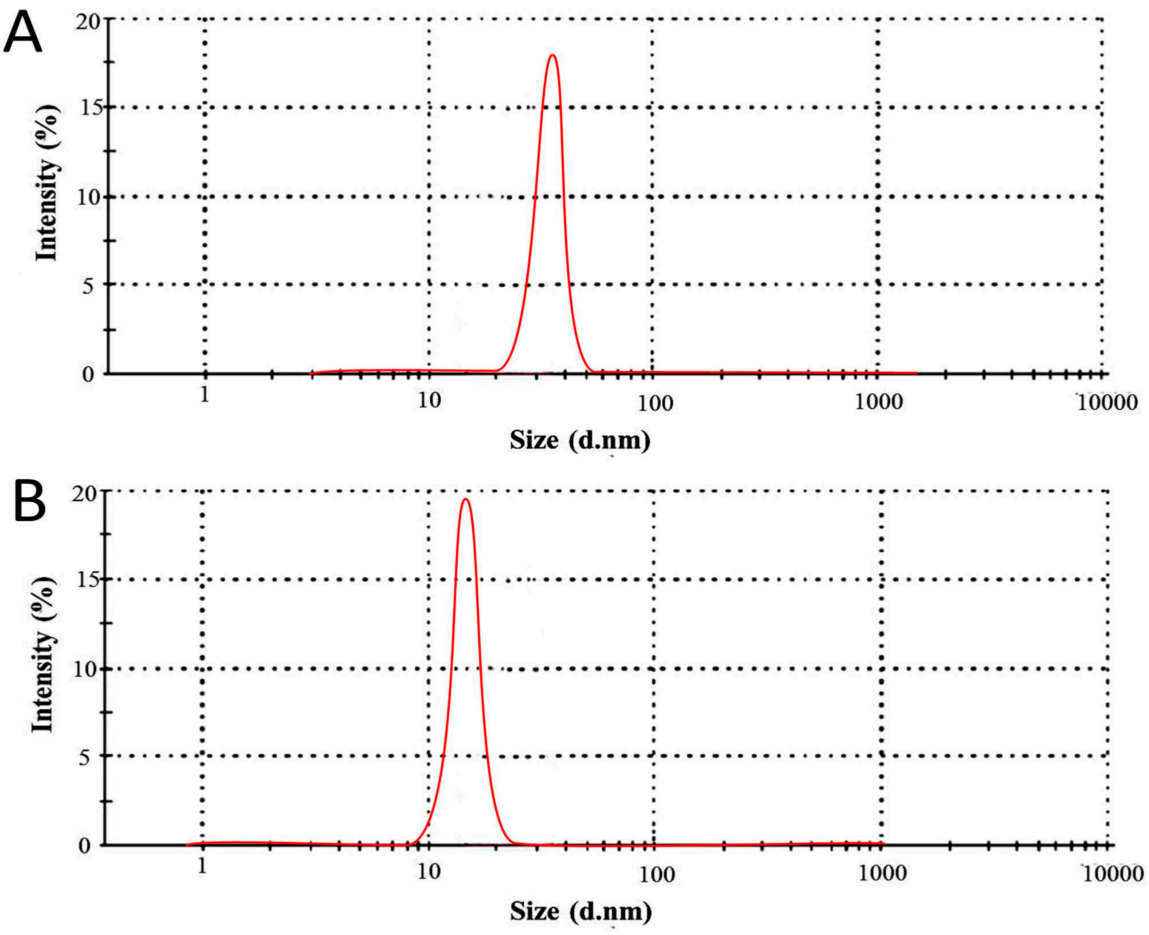

Supplementary material S3. Partial sequences of 5.8S ribosomal RNA, complete sequences of internal transcribed spacer 2, and partial sequences of large subunit ribosomal RNA gene for the *Fusarium verticillioides* isolate.

>OP870085.1 *Fusarium verticillioides* isolate ARC2 small subunit ribosomal RNA gene, partial sequence; internal transcribed spacer 1, 5.8S ribosomal RNA gene, and internal transcribed spacer 2, complete sequence; and large subunit ribosomal RNA gene, partial sequence

```
>Seq1
CTCCGTAGGTGAACCTGCGGAGGGATCATTACCGAGTTTACAACTCCCAAACCCCTGTGAACATACCAAT
TGTTGCCTCGGCGGATCAGCCCGCTCCCGGTAAAACGGGACGGCCCGCCAGAGGACCCCTAAACTCTGTT
TCTATATGTAACTTCTGAGTAAAACCATAAATAAATCAAAACTTTCAACAACGGATCTCTTGGTTCTGGC
ATCGATGAAKAMCKCCARMAAAATGCGATAAGTAATGTGAATTGCAGAATTCAGTGAATCATCGAATCTT
TGAACGCACATTGCGCCCGCCAGTATTCTGGCGGGCATGCCTGTTCGAGCGTCATTTCAACCCTCAAGCC
CAGCTTGGTGTTGGGACTCGCGAGTCAAATCGCGTTCCCCAAATTGATTGGCGGTCACGTCGAGCTTCCA
TAGCGTAGTAGTAAAACCCTCGTTACTGGTAATCGTCGCGGCCACGCCGTTAAACCCCAACTTCTGAATG
TTTACCTCGGATCAGGTAGGAATACCCGCTGAACTTAAGCATATCAATAACGGAGGA
```
